# Assessment of breathing in cardiac arrest: a randomised controlled trial of three teaching methods among laypersons

**DOI:** 10.1186/s12873-021-00513-4

**Published:** 2021-10-09

**Authors:** Niklas Breindahl, Anders Granholm, Theo Walther Jensen, Annette Kjær Ersbøll, Helge Myklebust, Freddy Lippert, Anne Lippert

**Affiliations:** 1 Copenhagen Academy for Medical Education and Simulation, Centre for HR&U, Borgmester Ib Juuls Vej 1, 2730 Herlev, Capital Region of Denmark Denmark; 2 Department of Intensive Care, Copenhagen University Hospital – Rigshospitalet, Copenhagen, Denmark; 3 Copenhagen Emergency Medical Services, Copenhagen, Denmark; 4 National Institute of Public Health, University of Southern Denmark, Copenhagen, Denmark; 5 Laerdal Medical, Stavanger, Norway; 6 Faculty of Health and Medical Sciences, University of Copenhagen, Copenhagen, Denmark

**Keywords:** Agonal breathing, Abnormal breathing, Gasping, Breathing patterns, Breathing assessment, Cardiac arrest, Simulation, Randomised controlled trial, Education, Basic life support

## Abstract

**Background:**

The aim of this trial was to compare a video- and a simulation-based teaching method to the conventional lecture-based method, hypothesizing that the video- and simulation-based teaching methods would lead to improved recognition of breathing patterns during cardiac arrest.

**Methods:**

In this Danish, investigator-initiated, stratified, randomised controlled trial, adult laypersons (university students, military conscripts and elderly retirees) participating in European Resuscitation Council Basic Life Support courses were randomised to receive teaching on how to recognise breathing patterns using a lecture- (usual practice), a video-, or a simulation-based teaching method. The primary outcome was recognition of breathing patterns in nine videos of actors simulating normal breathing, no breathing, and agonal breathing (three of each). We analysed outcomes using logistic regression models and present results as odds ratios (ORs) with 95% confidence intervals (CIs) and *P*-values from likelihood ratio tests.

**Results:**

One hundred fifty-three participants were included in the analyses from February 2, 2018 through May 21, 2019 and recognition of breathing patterns was statistically significantly different between the teaching methods (*P* = 0.013). Compared to lecture-based teaching (83% correct answers), both video- (90% correct answers; OR 1.77, 95% CI: 1.19–2.64) and simulation-based teaching (88% correct answers; OR 1.48; 95% CI: 1.01–2.17) led to significantly more correct answers. Video-based teaching was not statistically significantly different compared to simulation-based teaching (OR 1.20; 95% CI: 0.78–1.83).

**Conclusion:**

Video- and simulation-based teaching methods led to improved recognition of breathing patterns among laypersons participating in adult Basic Life Support courses compared to the conventional lecture-based teaching method.

**Supplementary Information:**

The online version contains supplementary material available at 10.1186/s12873-021-00513-4.

## Introduction

Agonal breaths are irregular and slow rasping respirations, frequently with a characteristic snoring sound as if the patient is gasping for air [1]. It is present in approximately 40 to 60% of victims during the first minutes of cardiac arrest [2–4]. The importance of early recognition of agonal breathing in out-of-hospital-cardiac-arrest (OHCA) has been emphasised in international guidelines during the past 15 years [5–8] due to increased survival rates if responded to as a sign of cardiac arrest [2, 9–16]. However, recognition of agonal breathing is difficult and frequently delays recognition of cardiac arrest [13, 17–25]. Teaching laypersons how to recognise agonal breathing is challenging [26]. Even trained laypersons certified as European Resuscitation Council (ERC) Basic Life Support (BLS) instructors only recognise agonal breathing in 61% of cases [26]. The development of more effective methods for teaching laypersons how to recognise agonal breathing thus has the potential to improve outcomes in patients with cardiac arrest initially presenting with agonal breathing.

The aim of this randomised controlled trial was to compare a video- and a simulation-based teaching method to the conventional lecture-based teaching method in ERC BLS courses on laypersons recognition of breathing patterns. We hypothesized that video- and simulation-based teaching methods would lead to improved recognition of breathing patterns.

## Materials and methods

### Trial design and oversight

We conducted a Danish, investigator-initiated, stratified, randomised controlled trial comparing two new teaching methods (video- and simulation-based) with the conventional lecture-based method on adult BLS course participants’ ability to recognise different breathing patterns. Participants and investigators were not blinded, except for the trial statistician (AKE) who conducted the pre-planned analyses blinded to the intervention groups.

Participants were randomly assigned to the three groups in a 1:1:1-ratio using computer-generated random allocation sequences with permuted blocks of varying sizes (three and six) stratified by participant type (see section Participants) [27]. Allocation concealment was ensured through the use of sequentially numbered, opaque, sealed envelopes. A person, not involved in any other aspect of the study, prepared both the randomisation sequences and the sealed envelopes.

The Committee on Health Research Ethics in the Capital Region of Denmark waived the need for ethical approval, as this was a teaching project (journal number: 17021633). All methods and experiments were performed in accordance with relevant guidelines and regulations. Participation in the trial was optional and voluntary and all participants gave written informed consent, which could be withdrawn without explanation at any time. The trial was approved by the Danish Data Protection Agency (journal number HGH-2017-131, I-Suite number: 06089). The trial was not publicly registered, as this is not mandatory for studies assessing teaching interventions in healthy volunteers. The trial was conducted in accordance with a pre-specified protocol and statistical analysis plan (available in Danish from the corresponding author upon request). There were no changes to trial outcomes after the trial commenced. The trial is reported in compliance with the Consolidated Standards of Reporting Trials (CONSORT) Statement [28] (completed checklist included in the Additional file 1).

### Participants

Three types of adult participants (≥18 years) were included: 1) university students (medical students excluded) from the University of Copenhagen, who had applied for a course through the organisation Student2Student (a voluntary organisation of medical students who teach BLS courses to non-medical university students); 2) conscripts in the Danish Military; and 3) retired elderly people who had applied for a BLS course through the Danish foundation TrygFonden. Participants were excluded if they had ever studied to become a health care professional (paramedic, nurse, physician, or other). The only additional criterion was a satisfactory continuous assessment at an ERC BLS course (4 h) where enrolment took place through the three organisations listed above. All BLS courses were provided free of charge. Participants received a reimbursement of 200 DKK (approximately €27) for their participation.

### BLS courses and interventions

Agonal breathing was not mentioned during the 4-h ERC BLS course and was only taught as part of the intervention according to the randomisation. After the course, participants were randomised to receive lecture-based, video-based or simulation-based teaching on how to recognise agonal breathing. All teaching sessions lasted approximately 2 min. The lecture group was taught using the ERC BLS 2015 course materials (a single slide in a lecture) and teaching methods according to ERC Guidelines for Resuscitation 2015 Section 2: Adult basic life support and automated external defibrillation [7]. The video group saw a video in plenary with text and verbal explanation and examples of agonal breathing (see Additional file) using recordings of HM simulating agonal breathing (originally developed for another purpose by Laerdal Medical, Stavanger, Norway; used with permission). In order to standardise the teaching methods, the instructor was not allowed to answer questions or to give examples on agonal breathing in the lecture group or video group. The participants in the simulation group simulated agonal breathing individually with the instructor. The instructor explained and gave some examples on agonal breathing the first 30 s. The following 90 s, the participants simulated with feedback from the instructor. All three interventions are described in more detail in the Additional file.

To minimise differences between the three ERC BLS instructors (NB, AG, and TWJ) responsible for the interventions, they all followed a detailed instructions manual on what to say and how to interact with the participants in all three groups (see Additional file 1). Participants were not allowed to interact with each other between the intervention and the test.

### Testing

Testing was performed immediately after the course using nine different videos of actors simulating normal breathing, no breathing and agonal breathing (three videos of each). The study group and a study investigator (AL) with extensive experience in medical teaching and BLS courses selected specific video clips for maximum clarity of various aspects of normal and agonal breathing. Videos consisted of 2 female and 7 male actors, all 40–60 years old and wearing T-shirts with high necks to avoid visualisation of the carotid artery while simulating breathing patterns. The videos lasted 30 s each and showed a side profile of a simulated patient’s head and torso. All videos were edited to give the actors a pale colour and to hide their carotid pulse. Three experienced paramedics (see acknowledgements) not otherwise involved in the study or the video production classified the videos with a 100% concordance to the intended categories. For the test, participants were informed that the persons in the videos were unconscious and had patent airways and watched all nine videos in the same order (see Additional file 1). After each video, participants had 10 s to classify the breathing pattern as either: 1) normal breathing, 2) no breathing, 3) agonal breathing, or 4) “do not know”, in order to minimise random guesses. Option 4) “do not know” was also used if participants did not answer or answered after the 10 s between each video, which was considered an incorrect response in the analyses. Participants were under observation by one of the instructors during the entire test. Each video could only be seen once, and participants were not allowed to communicate during the test. The testing procedures are fully accounted for in the Additional file 1. Data were registered on paper-based forms, and all data were electronically entered twice and compared to ensure data quality and avoid transcription errors.

### Outcomes

The primary outcome was the participants’ overall ability to recognise all three breathing patterns in terms of the total number of correct breathing assessments out of all nine videos. The secondary outcomes were the participants’ ability to recognise each breathing pattern (normal breathing, no breathing, and agonal breathing) in three videos of each pattern.

### Statistical analyses and sample size

The required sample size was calculated using a 5% significance level and 80% power. Assuming that participants in the lecture group would have a mean number of correct answers of 4–5 out of 9 videos, and that the participants in the video group and the simulation group would have a mean of 2–3 correct answers more than the participants in the lecture group, 42–48 participants were needed in each group. This assumption was made according to our best estimate as instructors teaching laypersons using the lecture method. To ensure an adequate sample size and allow for possible withdrawals of consent, we planned to include 50 participants in each intervention group. We offered inclusion to all participants present at each course (maximum of six participants per course) until we reached a minimum of 150 included participants in total. No interim analyses were planned or performed. There were no missing data and thus no need for imputation of missing data.

Baseline characteristics, including gender, age, education and previous BLS experience with an estimate of years since last BLS course, are presented separately for each group and for the total trial population. Categorical variables are presented as frequencies (counts and percentages) and numerical variables as medians with interquartile ranges (IQR) due to non-normality (inspected using histograms and Q-Q-plots), and post-hoc also as categorical variables due to non-normality.

Primary and secondary outcomes are presented as counts and percentages of correct answers and analysed using logistic regression models adjusted for participant type (as described above). The effect of teaching method on recognition of breathing patterns was tested using likelihood ratio tests, and the relative effects of teaching methods were compared using odds ratios (ORs) with 95% confidence intervals (CIs) calculated using the Wald method. In addition to the pre-planned analyses, we conducted a post-hoc analysis of the primary outcome. This analysis was conducted using a logistic regression model including the main effect of teaching method and participant type and the interaction between teaching method and participant type. A likelihood ratio test of the interaction was conducted. The relative effects of teaching methods within participant types were compared using odds ratios (ORs) with 95% confidence intervals (CIs) calculated using the Wald method. We considered *P*-values < 0.05 and 95% CIs for ORs not including 1.00 as statistically significant. All analyses were performed using SAS version 9.4 (SAS Institute, Cary, NC, USA), with logistic regression analyses performed using the GENMOD procedure.

## Results

### Trial population

**Fig. 1:**
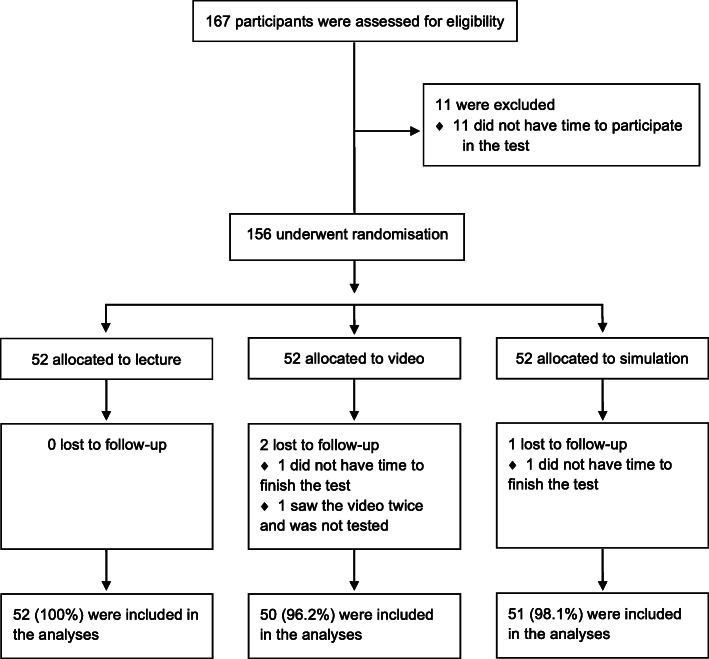
CONSORT inclusion flowchart of screening, randomisation and participation. Legend: 167 course participants were assessed for eligibility. Eleven course participants were excluded before randomisation, as they did not have time to participate in the test. The remaining 156 participants underwent randomisation and 52 were allocated to each intervention group. Two participants had to leave before the test after the randomisation and one participant in the video group saw the video twice and was erroneously not tested. A total of 153 participants completed the test and were included in the analyses

From February 2, 2018 through May 21, 2019, we assessed 167 course participants enrolled in an ERC BLS course through the organisations listed above for eligibility (Fig. 1). All course participants had a satisfactory continuous assessment, and none of the participants had ever studied to become a health care professional. We excluded 11 course participants before randomisation, as they did not have time to participate in the test. The remaining 156 participants underwent randomisation and 52 were allocated to each intervention group. Two participants had to leave before the test (one in the video group and one in the simulation group), and one participant in the video group saw the video twice and was erroneously not tested. These three participants were excluded from all analyses, including analyses of baseline data. A total of 153 participants completed the test and were included in the analyses (Fig. 1). The baseline characteristics of the participants were largely similar in the three groups (Table 1). No baseline or outcome data were missing for these participants.

**Table 1:** Characteristics of the participants at baseline

Characteristic	Lecture (*N* = 52)	Video (*N* = 50)	Simulation (*N* = 51)	Total (*N* = 153)
Participant types, N (%)				
University students	15 (28.9)	15 (30.0)	17 (33.3)	47 (30.7)
Military conscripts	18 (34.6)	19 (38.0)	17 (33.3)	54 (35.3)
Elderly retirees	19 (36.5)	16 (32.0)	17 (33.3)	52 (34.0)
Male, N (%)	30 (57.7)	32 (64.0)	29 (56.9)	91 (59.5)
Age, median (IQR)	26.5 (21.0; 69.5)	24.0 (20.0; 67.0)	24.0 (21.0; 70.0)	26.0 (21.0; 69.0)
Age, N (%)				
≤ 20 years	10 (19.2)	14 (28.00)	11 (21.6)	35 (22.9)
21–30 years	20 (38.5)	19 (38.00)	21 (41.2)	60 (39.2)
31–40 years	3 (5.8)	1 (2.00)	1 (1.9)	5 (3.3)
51–60 years	0 (0.00)	0 (0.00)	1 (1.9)	1 (0.7)
61–70 years	8 (15.4)	9 (18.00)	5 (9.8)	22 (14.4)
> 70 years	11 (21.2)	7 (14.00)	12 (23.5)	30 (19.6)
Previous BLS course, N (%)	35 (67.3)	38 (76.0)	38 (74.5)	111 (72.5)
Years since last BLS course, median (IQR)	4.0 (2.0; 6.0)	2.5 (2.0; 5.0)	3.0 (2.0; 8.0)	3.0 (2.0; 6.0)
Years since, N (%)				
< 3 years	9 (17.3)	19 (38.0)	11 (21.6)	39 (25.5)
3–5 years	16 (30.8)	10 (20.0)	15 (29.4)	41 (26.8)
> 5 years	10 (19.2)	9 (18.0)	12 (23.5)	31 (20.3)
Never	17 (32.7)	12 (24.0)	13 (25.5)	42 (27.5)
Highest completed education, N (%)				
Lower secondary school	3 (5.8)	6 (12.0)	6 (11.8)	15 (9.8)
Adult vocational training	12 (23.1)	9 (18.0)	7 (13.7)	28 (18.3)
Upper secondary school	24 (46.2)	25 (50.0)	26 (51.0)	75 (49.0)
University College	4 (7.7)	6 (12.0)	7 (13.7)	17 (11.1)
University	8 (15.4)	4 (8.0)	5 (9.8)	17 (11.1)
Other	1 (1.9)	0 (0.0)	0 (0.0)	1 (0.7)

Baseline characteristics of the participants assigned to the three different teaching methods (lecture-, video- and simulation-based). Lower secondary school is the final part of the compulsory education in Denmark lasting 10 years, adult vocational training is mainly for low skilled and skilled workers on the labour market, upper secondary school (additional 2–3 years) is an admission requirement for university college and university. University College is offering medium-cycle programmes and university is offering long-cycle programmes

*Abbreviations*: *IQR* interquartile range, *BLS* Basic Life Support

### Primary outcome

**Fig. 2:**
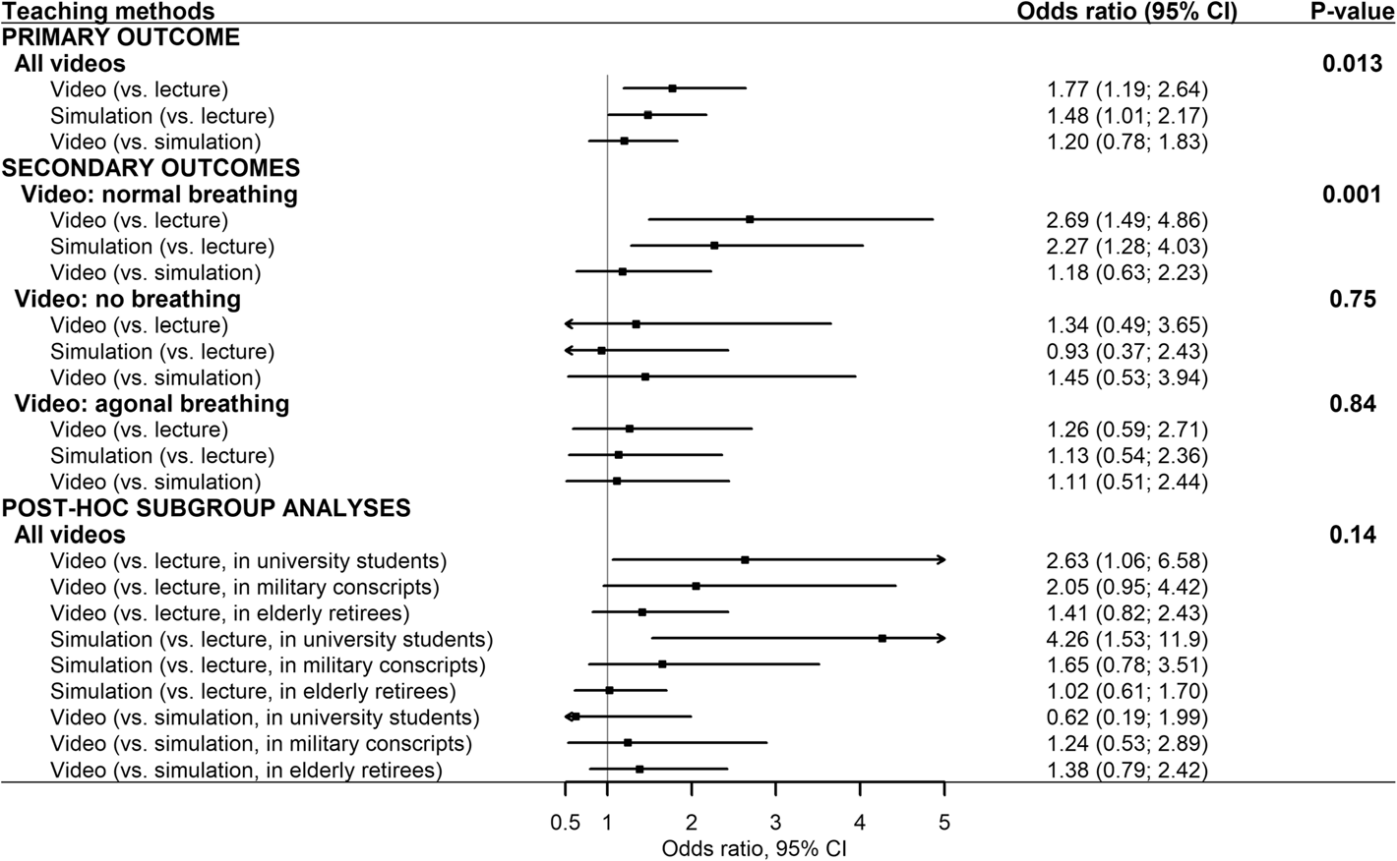
Forest plot with outcome measures and the post-hoc analysis of the primary outcome. Legend: The overall differences are presented as *P*-values calculated using likelihood ratio tests, the relative effects of teaching methods are presented as odds ratios with 95% confidence intervals (CIs) calculated using the Wald method

All results are presented in Table 2 and Fig. 2. The three teaching methods were overall significantly different (*P* = 0.013). Compared to lecture-based teaching (83% correct answers), both video- (OR 1.77, 95% CI: 1.19–2.64) and simulation-based teaching (OR 1.48; 95% CI: 1.01–2.17) led to significantly more correct answers (90 and 88%, respectively). Video-based teaching was not significantly different from simulation-based teaching (OR 1.20; 95% CI 0.78–1.83, simulation group as reference).

**Table 2:** Numbers and proportions of correct answers for primary and secondary outcomes

	Correct answers/number of answers (%)
PRIMARY OUTCOME	
All videos	
*Lecture*	389/468 (83%)
*Video*	405/450 (90%)
*Simulation*	405/459 (88%)
SECONDARY OUTCOMES	
Normal breathing	
*Lecture*	104/156 (67%)
*Video*	125/150 (83%)
*Simulation*	124/153 (81%)
No breathing	
*Lecture*	139/156 (89%)
*Video*	137/150 (91%)
*Simulation*	138/153 (90%)
Agonal breathing	
*Lecture*	146/156 (94%)
*Video*	143/150 (95%)
*Simulation*	143/153 (93%)

Total number and proportions of correct answers for primary outcome (recognition of breathing patterns) and secondary outcomes (recognition of each breathing pattern). Numbers are calculated as the total number of correct answers (for example, 52 participants were randomised to receive lecture-based teaching and watched a total of 9 videos for the primary outcome providing the possibility of 468 correct answers)

### Secondary outcomes

There was an overall statistically significant difference between the teaching methods regarding recognition of normal breathing (*P* = 0.001), but no significant differences for recognition of no breathing (*P* = 0.75) or agonal breathing (*P* = 0.84) (Table 2 and Fig. 2). Compared to lecture-based teaching (67% correct answers), both video- (OR 2.69, 95% CI: 1.49–4.86) and simulation-based teaching (OR 2.27, 95% CI: 1.28–4.03) led to significantly more correct classifications of normal breathing (83 and 81% correct answers, respectively). Video-based teaching was not significantly different compared to simulation-based teaching according to recognition of normal breathing (OR 1.18, 95% CI: 0.63–2.23, simulation group as reference).

### Post-hoc analysis

The analysis of differences between teaching methods within participant type was not statistically significant (*P* = 0.14); differences were statistically significant in university students when comparing video-based (95% correct answers, OR 2.63, 95% CI: 1.06–6.58) and simulation-based (97% correct answers, OR 4.26, 95% CI: 1.53–11.9) vs. lecture-based teaching (87% correct answers) (Table 3 and Fig. 2).

**Table 3:** Proportions of correct answers in the post-hoc analysis of the primary outcome

Post-hoc subgroup analysis	Correct answers/number of answers (%)
Lecture-based teaching	
*University students*	118/135 (87%)
*Military conscripts*	142/162 (88%)
*Elderly retirees*	129/171 (75%)
Video-based teaching	
*University students*	128/135 (95%)
*Military conscripts*	160/171 (94%)
*Elderly retirees*	117/144 (81%)
Simulation-based teaching	
*University students*	148/153 (97%)
*Military conscripts*	141/153 (92%)
*Elderly retirees*	116/153 (76%)

Total number and proportions of correct answers in the post-hoc subgroup analysis of the primary outcome (recognition of breathing patterns) investigating the effect of teaching method on the different subgroups (university students, military conscripts and elderly retirees). Numbers are calculated as the total number of correct answers (for example, 15 university students were randomised to receive lecture-based teaching and watched a total of 9 videos for the primary outcome providing the possibility of 135 correct answers)

## Discussion

Both video-based and simulation-based teaching methods led to a statistically significantly improved recognition of breathing patterns compared to conventional lecture-based teaching. Laypersons receiving the lecture-based intervention in our trial correctly identified normal breathing in 67% and no breathing in 89% of cases, similar to a study of Perkins et al from 2005, where 2nd year medical students correctly identified normal breathing and no breathing in 61 and 85% of the cases, respectively [26]. The results of this trial differ somewhat, as laypersons receiving the lecture-based intervention correctly identified agonal breathing 94% of cases, whereas 2nd year medical students trained as BLS instructors only identified agonal breathing correctly in 61% of cases in the previous study [26]. Perkins et al provided only 10 s of video for each breathing pattern, reflecting the maximum time recommended in current guidelines for breathing assessment [6]. To detect a difference between the breathing patterns and to ensure the participants’ ability to assess the regularity of breathing even at a low respiratory rate, we decided to give the participants 30 s to watch each breathing pattern and 10 s to make a decision afterwards. Consequently, recognition of agonal breathing in our trial may have been too easy. We fully support the current guidelines and recommend only 10 s of breathing assessment, yet spending longer time on this may reflect reality for laypersons initially perceiving the victim’s breathing as normal [25].

### Strengths

The strengths of our trial include the use of only three instructors, a detailed instructions manual limiting inter-instructor variations in interventions, pre-specification of the analyses conducted and no missing data for the included participants. Health-care professionals were excluded, and three different types of participants were included (university students, military conscripts and elderly retirees) to get a broad representation of laypersons. The university students and military conscripts mainly consisted of young adults in contrast to the elderly retirees and had a higher proportion of correct answers for every intervention in the post-hoc analysis. This could be due to their lowered sensory abilities and cognitive abilities making the test particularly difficult in this age group [29, 30]. Yet, even though the teaching materials and the test were not adapted for the elderly group, the teaching interventions had the same relative effect as on the two younger groups.

Although these three types of participants may not be representative for the heterogeneous population of laypersons worldwide, inclusion of only three types permitted us to stratify the trial and adjust our analyses accordingly, and thus minimise the influence of differences between different types of participants on our results.

Results from this trial indicate a higher ability to correctly classify breathing patterns during breathing assessment in cardiac arrest if laypersons are taught using video- or simulation-based teaching on ERC BLS courses. Video-based teaching may be superior to simulation-based teaching in several aspects. First, video-based teaching is not dependent on instructor skills and does not require specific instructor training. Second, it can serve to standardise and quality-assure the teaching between various instructors, course organisers and resuscitation councils worldwide. Third, it can be carried out in plenary, and therefore requires a significantly smaller amount of time per participant compared to simulation, where each participant needs to be involved in the simulation to ensure an optimal learning outcome. Thus, video-based teaching can be implemented in existing BLS courses without substantial increases in difficulty, time requirements or cost. Finally, videos may be made available to participants allowing them to revisit the material after the course, which may increase retention.

### Limitations

Our study has some limitations. First, the quality of the three teaching methods was evaluated using video recordings of actors simulating breathing types and not actual patients. However, using actual patients was not possible at that time and would have been ethically questionable. To ensure a high level of realism in the testing videos, we recorded numerous attempts at agonal breathing by many actors and chose the best versions of agonal breathing for testing purpose. However, a video of an actor may not be 100% in accordance with real life. Second, participants in our trial should only focus on breathing assessment and no subsequent treatment, which might have provided them with a greater ability to concentrate exclusively on the breathing assessment. Our testing videos do not take into account the complexity of breathing assessment in a real cardiac arrest where the bystander is highly affected by uncertainty and stress due to emotional attachment to the victim, continuous disturbances from various sensory input or input from team members and other bystanders or relatives. Third, immediate evaluation of agonal breathing after being taught how to recognise this could potentially bias the results and explain why participants recognised agonal breathing slightly better than no breathing. However, any other solution would have been very difficult to organise. Further, the trial was only adequately powered for the primary outcome with three times as many answers as the secondary outcomes. We did not find any statistically significant differences in two of three secondary outcomes (no breathing and agonal breathing), which may be explained by limited power, but could also be because the interventions led to smaller or no differences for these breathing patterns. In addition, we have not assessed retention or the effect on time to recognition in real cardiac arrest situations, and future studies are needed to investigate whether these improved teaching methods translate to improved practice. Finally, the analysis of differences between teaching methods within participant type was not statistically significant. As the study was not powered to detect subgroup differences, this could either be because of lack of power, or because the effects of different teaching methods are similar within participant type.

## Conclusion

Video-based and simulation-based teaching methods led to improved recognition of breathing patterns in adult BLS courses for lay persons compared to the conventional lecture-based teaching method.

## Supplementary Information

**Additional file 1.**

## Data Availability

The datasets used and/or analysed during the current study are available from the corresponding author on reasonable request.

## Abbreviations

BLS: Basic life support
CI: Confidence interval
CONSORT: Consolidated Standards of Reporting Trials
ERC: European Resuscitation Council
OHCA: Out-of-hospital-cardiac-arrest
OR: Odds ratio

## References

[1] Riou M, Ball S, Williams TA, Whiteside A, Cameron P, Fatovich DM, Perkins GD, Smith K, Bray J, Inoue M, O’Halloran KL, Bailey P, Brink D, Finn J (2018). ‘She’s sort of breathing’: What linguistic factors determine call-taker recognition of agonal breathing in emergency calls for cardiac arrest?. Resuscitation..

[2] Bobrow BJ, Zuercher M, Ewy GA, Clark L, Chikani V, Donahue D, Sanders AB, Hilwig RW, Berg RA, Kern KB (2008). Gasping during cardiac arrest in humans is frequent and associated with improved survival. Circulation..

[3] Clark JJ, Larsen MP, Culley LL, Graves JR, Eisenberg MS (1992). Incidence of agonal respirations in sudden cardiac arrest. Ann Emerg Med.

[4] Fukushima H, Imanishi M, Iwami T, Seki T, Kawai Y, Norimoto K, Urisono Y, Hata M, Nishio K, Saeki K, Kurumatani N, Okuchi K (2015). Abnormal breathing of sudden cardiac arrest victims described by laypersons and its association with emergency medical service dispatcher-assisted cardiopulmonary resuscitation instruction. Emerg Med J.

[5] Handley AJ, Koster R, Monsieurs K, Perkins GD, Davies S, Bossaert L (2005). European resuscitation council guidelines for resuscitation 2005. Resuscitation..

[6] Koster RW, Baubin MA, Bossaert LL, Caballero A, Cassan P, Castrén M, Granja C, Handley AJ, Monsieurs KG, Perkins GD, Raffay V, Sandroni C (2010). European resuscitation council guidelines for resuscitation 2010 section 2. Adult basic life support and use of automated external defibrillators. Resuscitation..

[7] Perkins GD, Handley AJ, Koster RW, Castrén M, Smyth MA, Olasveengen T, Monsieurs KG, Raffay V, Gräsner JT, Wenzel V, Ristagno G, Soar J, Bossaert LL, Caballero A, Cassan P, Granja C, Sandroni C, Zideman DA, Nolan JP, Maconochie I, Greif R (2015). European resuscitation council guidelines for resuscitation 2015. Resuscitation..

[8] Panchal AR, Bartos JA, Cabañas JG, Donnino MW, Drennan IR, Hirsch KG (2020). Part 3: adult basic and advanced life support: 2020 American Heart Association guidelines for cardiopulmonary resuscitation and emergency cardiovascular care. Circulation..

[9] Debaty G, Labarere J, Frascone RJ, Wayne MA, Swor RA, Mahoney BD, Domeier RM, Olinger ML, O’Neil BJ, Yannopoulos D, Aufderheide TP, Lurie KG (2017). Long-term prognostic value of gasping during out-of-hospital cardiac arrest. J Am Coll Cardiol.

[10] Ewy GA, Armstrong PW (2017). A natural biomarker deserving attention. J Am Coll Cardiol.

[11] Zhang Q, Liu B, Qi Z, Li C (2018). Prognostic value of gasping for short and long outcomes during out-of-hospital cardiac arrest: an updated systematic review and meta-analysis. Scand J Trauma Resusc Emerg Med.

[12] Knor J, Seblova J, Skulec R, Seblova D, Malek J (2018). The presence of gasping predicts long-term survival in out-of-hospital cardiac arrest patients. Biomed Pap.

[13] Fukushima H, Panczyk M, Hu C, Dameff C, Chikani V, Vadeboncoeur T, et al. Description of abnormal breathing is associated with improved outcomes and delayed telephone cardiopulmonary resuscitation instructions. J Am Heart Assoc. 2017;6(9). 10.1161/JAHA.116.005058. 10.1161/JAHA.116.005058 PMC5634247 28851728

[14] Wissenberg M, Lippert FK, Folke F, Weeke P, Hansen CM, Christensen EF, Jans H, Hansen PA, Lang-Jensen T, Olesen JB, Lindhardsen J, Fosbol EL, Nielsen SL, Gislason GH, Kober L, Torp-Pedersen C (2013). Association of national initiatives to improve cardiac arrest management with rates of bystander intervention and patient survival after out-of-hospital cardiac arrest. JAMA..

[15] Berdowski J, Beekhuis F, Zwinderman AH, Tijssen JGP, Koster RW (2009). Importance of the first link: description and recognition of an out-of-hospital cardiac arrest in an emergency call. Circulation..

[16] Lewis M, Stubbs BA, Eisenberg MS (2013). Dispatcher-assisted cardiopulmonary resuscitation: time to identify cardiac arrest and deliver chest compression instructions. Circulation..

[17] Hardeland C, Sunde K, Ramsdal H, Hebbert SR, Soilammi L, Westmark F, Nordum F, Hansen AE, Steen-Hansen JE, Olasveengen TM (2016). Factors impacting upon timely and adequate allocation of prehospital medical assistance and resources to cardiac arrest patients. Resuscitation..

[18] Brinkrolf P, Metelmann B, Scharte C, Zarbock A, Hahnenkamp K, Bohn A (2018). Bystander-witnessed cardiac arrest is associated with reported agonal breathing and leads to less frequent bystander CPR. Resuscitation..

[19] Breckwoldt J, Schloesser S, Arntz H-R (2009). Perceptions of collapse and assessment of cardiac arrest by bystanders of out-of-hospital cardiac arrest (OOHCA). Resuscitation..

[20] Vaillancourt C, Charette ML, Bohm K, Dunford J, Castrén M (2011). In out-of-hospital cardiac arrest patients, does the description of any specific symptoms to the emergency medical dispatcher improve the accuracy of the diagnosis of cardiac arrest: a systematic review of the literature. Resuscitation..

[21] Hauff SR, Rea TD, Culley LL, Kerry F, Becker L, Eisenberg MS (2003). Factors impeding dispatcher-assisted telephone cardiopulmonary resuscitation. Ann Emerg Med.

[22] Eisenberg MS (2006). Incidence and significance of gasping or agonal respirations in cardiac arrest patients. Curr Opin Crit Care.

[23] Rea TD (2005). Agonal respirations during cardiac arrest. Curr Opin Crit Care.

[24] Dami F, Heymann E, Pasquier M, Fuchs V, Carron P-N, Hugli O (2015). Time to identify cardiac arrest and provide dispatch-assisted cardio-pulmonary resuscitation in a criteria-based dispatch system. Resuscitation..

[25] Travers S, Jost D, Gillard Y, Lanoë V, Bignand M, Domanski L, Tourtier JP, Paris Fire Brigade Cardiac Arrest Work Group (2014). Out-of-hospital cardiac arrest phone detection: those who most need chest compressions are the most difficult to recognize. Resuscitation..

[26] Perkins GD, Stephenson B, Hulme J, Monsieurs KG (2005). Birmingham assessment of breathing study (BABS). Resuscitation..

[27] Altman DG, Bland JM (1999). Statistics notes: how to randomise. BMJ..

[28] Schulz KF, Altman DG, Moher D, CONSORT Group (2010). CONSORT 2010 Statement: Updated guidelines for reporting parallel group randomised trials. J Clin Epidemiol.

[29] Worcester MI, Loustau A, O’Connor K (1990). Tailoring teaching to the elderly in home care. Home Health Care Serv Q.

[30] Stevens B (2003). How seniors learn. Issue Brief Cent Medicare Educ.

