# Supplementary material for: Assessment of breathing in cardiac arrest: a randomised controlled trial of three teaching methods among laypersons
Source: BMC Emerg Med. 2021 Oct 9;21:114. doi: 10.1186/s12873-021-00513-4 (PMC8502323; doi:10.1186/s12873-021-00513-4)
Supplement: Supplementary file 1 — Additional file 1. [file 12873_2021_513_MOESM1_ESM.docx]

**Assessment of breathing in cardiac arrest:**

**a randomised controlled trial of three teaching methods among laypersons**

**Additional file 1**

Niklas Breindahl^1,2*^, Anders Granholm^2,3^, Theo Walther Jensen^2,4^, Annette Kjær Ersbøll^5^,

Helge Myklebust^6^, Freddy Lippert^4^, Anne Lippert^2^

^1^ Faculty of Health and Medical Sciences, University of Copenhagen, Denmark

^2^ Copenhagen Academy for Medical Education and Simulation (CAMES), Copenhagen University Hospital – Herlev Hospital, Denmark

^3^ Department of Intensive Care, Copenhagen University Hospital – Rigshospitalet, Copenhagen, Denmark

^4^ Emergency Medical Services, Capital Region, Denmark

^5^ National Institute of Public Health, University of Southern Denmark, Odense, Denmark

^6^ Laerdal Medical, Stavanger, Norway

**Corresponding author:**

*Niklas Breindahl, medical student*

*Copenhagen Academy for Medical Education and Simulation (CAMES), Copenhagen University*

*Hospital - Herlev Hospital*

*Borgmester Ib Juuls Vej 1, 2730 Herlev*

*Denmark*

*E-mail:* [*niklas.breindahl@gmail.com*](mailto:niklas.breindahl@gmail.com)

TABLE OF CONTENTS

Additional methodological details 3

1 | Instructors 3

2 | Teaching methods 3

*2.1 | Lecture-based teaching (standard ERC BLS course)* 3

*2.2 | Video-based teaching* 4

*2.3 | Simulation-based teaching* 4

3 | Testing 4

4 | CONSORT checklist 5

Additional tables and figures 6

Table A1. Modified ERC BLS 4-hour course programme (2015 guidelines). 6

Table A2. Script for teaching video. 7

Table A3. Script for test video. 8

Table A4. Completed CONSORT checklist. 9

# Additional methodological details

## 1 | Instructors

All instructors are certified European Resuscitation Council (ERC) Basic Life Support (BLS) instructors. All three were involved in the study design.

## 2 | Teaching methods

All teaching methods were implemented in the existing ERC BLS 4-hour course, approved by the Danish Resuscitation Council (course programme presented in Additional Table A1). We estimated the mean time spent on teaching agonal breathing during ERC 4-hour BLS courses to be approximately two minutes, and chose this as the approximate time limit for all teaching methods. For simulation-based teaching, which was done individually, approximately two minutes were used per participant.

To minimise differences between the three instructors responsible for the interventions, all instructors followed an instructions manual on what to say and how to interact with the participants. The participants were not allowed to interact with each other between the intervention and the final test, which took place right after the termination of the ERC BLS course. Agonal breathing was never explained during the rest of the course.

### *2.1 | Lecture-based teaching (standard ERC BLS course)*

This group acted as the control group, reflecting standard teaching practice. They received a short lecture (approximately two minutes) in plenary using the standardised ERC slideshow containing the following text (proprietary of ERC; www.erc.edu):

Title: “*Breathing*”.
Subtitle: “*Abnormal breathing*”.
Text in bullet points:

- “*Occurs shortly after the heart stops in up to 40% of cardiac arrests”*
- *“Described as barely, heavy, noisy or gasping breathing”*
- *“Recognise as a sign of cardiac arrest”* (in red font colour, edited).

The lecture followed a standardised script (translated from Danish):

“*You are now capable of starting cardiopulmonary resuscitation in case you have an unresponsive person who is not breathing normally – the clinical definition of a cardiac arrest. You will now be taught how to recognise various clinical expressions of agonal breathing. Agonal breathing occurs in up to 40% of cardiac arrests and can have many different presentations. It can be described as slow and deep breaths, as if the victim is barely breathing, frequently with a characteristic snoring sound as if the breathing is heavy, noisy or gasping. It is a sign of oxygen deprivation that occurs in the first minutes after cardiac arrest and should be recognised as a sign of cardiac arrest. The presence of agonal breathing can be erroneously interpreted as evidence that there is normal breathing. A victim who has no breathing is also not breathing normally.*”

The instructors were not allowed to answer questions or to give examples illustrating agonal breathing.

### *2.2 | Video-based teaching*

This group watched a video (two and a half minutes excluding introduction and ending) in plenary with visual examples on agonal breathing accompanied by written and verbal explanation (Additional Table A2). The instructor was present in the room during the entire video and was not allowed to answer questions or to give examples on agonal breathing.

The original Danish teaching video is available online: <https://youtu.be/oVRrh-ZyCk0>

The translated English teaching video is available online: <https://youtu.be/lVprCOe6mPQ>

### *2.3 | Simulation-based teaching*

The participants in this group individually received a short 30-second introduction before individually simulating agonal breathing with the instructor while receiving feedback for approximately 90 seconds (2 minutes in total). The simulation was introduced using the following script:

“*You are now capable of starting cardiopulmonary resuscitation in case you have an unresponsive person who is not breathing normally, which is the definition of a cardiac arrest. You will now be simulating various examples of agonal breathing together with me. I will start with a demonstration; afterwards it is your turn. You can ask questions, when I have given the first demonstration. Do you have any questions?”*

*“The clinical presentation of agonal breathing can vary: it can be slow, irregular and with deep breaths as if the victim is barely breathing, but the victim is NOT breathing normally. You will often hear a characteristic noisy, snoring or gasping sound from the victim. Sometimes you will observe characteristic reflective movements of the mouth and tongue. It can for example look like this [examples].* *Now it is your turn to simulate.”*

## 3 | Testing

The final test consisted of a 60-second introduction (Additional Table A3) followed by a total of nine videos of actors simulating various breathing patterns for 30 seconds each. Videos consisted of 2 female and 7 male actors, all 40-60 years old and wearing T-shirts with high necks to avoid visualisation of the carotid artery while simulating breathing patterns. Each person was unresponsive, and the participants were told to assume that they had already assessed that the person had a patent airway, as this was not assessed in the videos. Nine videos of normal breathing, agonal breathing and no breathing (three of each) were shown in the following order: 1) normal breathing, 2) no breathing, 3) agonal breathing, 4) no breathing, 5) agonal breathing, 6) normal breathing, 7) normal breathing, 8) no breathing, 9) agonal breathing. The participants had 10 seconds after each video to choose what kind of breathing pattern they observed from the following four options: 1) normal breathing, 2) agonal breathing, 3) no breathing, 4) do not know. If they did not choose within 10 seconds, it was registered as “do not know”, which also counted as an incorrect answer. Each video could only be watched once, and participants could not communicate during the test.

The original Danish testing videos are available online: <https://youtu.be/gQADhKFGLeg> (with translations of the speak/text provided in Additional Table A3).

## 4 | CONSORT checklist

A completed Consolidated Standards of Reporting Trials (CONSORT) checklist (www.consort-statement.org) is included in Additional Table A4.

# Additional tables and figures

## Table A1. Modified ERC BLS 4-hour course programme (2015 guidelines).

| **Duration** | **Activity** | **Method** |
| --- | --- | --- |
|  | Registration |  |
| 15 min | Introduction and welcome to the BLS with the use of an AED course | Plenary |
| *10 min* | *Oral information on the study**  *Collection of informed consent forms from the participants* | *Plenary* |
| 10 min | **BLS demonstration until AED arrives:**  Stage 1 (real-time demo)  Stage 2 (commentary) | Plenary |
| 70 min | Stage 3 (instructor demonstration with candidates’ commentary)  Stage 4 (practice until AED arrives) | Groups |
| *25 min* | ***Randomisation, interventions and break:***  *Break and randomisation/interventions (three different teaching methods)* | *Groups/individually* |
| 15 min | **BLS demonstration with AED:**  Stage 1 (real-time demo)  Stage 2 (commentary) | Plenary |
| 80 min | Stage 3 (instructor demonstration with candidates’ commentary)  Stage 4 (practice with AED) | Groups |
| 10 min | Break | Plenary |
| 10 min | **Recovery Position:**  Stage 1 (real-time demo)  Stage 2 (commentary) | Plenary |
| 10 min | Stage 3 (instructor demonstration with candidates’ commentary)  Stage 4 (practice) | Groups |
| 15 min | **Foreign body airway obstruction – choking:**  Demonstration and practice | Groups |
| 5 min | Questions and answers  Summary and closure | Plenary |
| *20 min* | ***Testing*** | *Groups/individually* |

* Participants received written information on the course and study before attending.
Abbreviations: BLS: Basic Life Support; AED: automated external defibrillator. Italic writing marks the implementation of study protocol and is not a part of the original course programme.

## Table A2. Script for teaching video.

The actor was wearing a blue polo shirt with a high neck to avoid visualisation of the pulse in the carotid artery.

| **Duration (seconds)** | **Voice-over** |
| --- | --- |
| 15 | *A person who is unresponsive and not breathing normally is in cardiac arrest, and bystanders should immediately start cardiopulmonary resuscitation. Agonal breathing or gasping may be present in up to 40% of victims in the first minutes after cardiac arrest and can erroneously be interpreted as signs of normal circulation. As a bystander, it is therefore important to be able to recognise agonal breathing and act accordingly.* |
| 20 | *The person in this video is acting.*  *The person is unresponsive. In this video we suppose that the person has a patent airway. The person in this video is illustrating some examples of agonal breathing.* |
| 15 | *Agonal breathing can be described as slow and deep breaths or gasps, but the victim is NOT breathing normally.* |
| 35 | *You will often hear a characteristic snoring, noisy sound from the victim.* |
| 35 | *The mouth and tongue are sometimes moving, almost like a stranded fish gasping for air.* |
| 50 | *The pause between each breath can sometimes be long.*  *Sometimes it can be very short.* |
| 15 | *Thank you for your attention. It is very important that you understand that these are only a few examples of agonal breathing. The video has now come to its end.* |

## Table A3. Script for test video.

| **Duration (seconds)** | **Explanation as text on black screen** |
| --- | --- |
| 60 | *You are now capable of starting cardiopulmonary resuscitation in case you have an unresponsive person who is not breathing normally, and we would like your help to evaluate the effect of the course.*  *You are about to assess nine persons on video. Each video takes 30 seconds.*  *Each person is unresponsive.*  *Each person has a patent airway.*  *When you have watched one video, please answer the question: “What kind of breathing pattern did you observe?” You may choose between the following four options:*   1. *Normal breathing* 2. *Agonal breathing* 3. *No breathing* 4. *Do not know*   *You have 10 seconds to choose, before the next video will start automatically.*  *If you do not choose within 10 seconds, it counts as “do not know”.*  *The test will start now.* |

## Table A4. Completed CONSORT checklist.


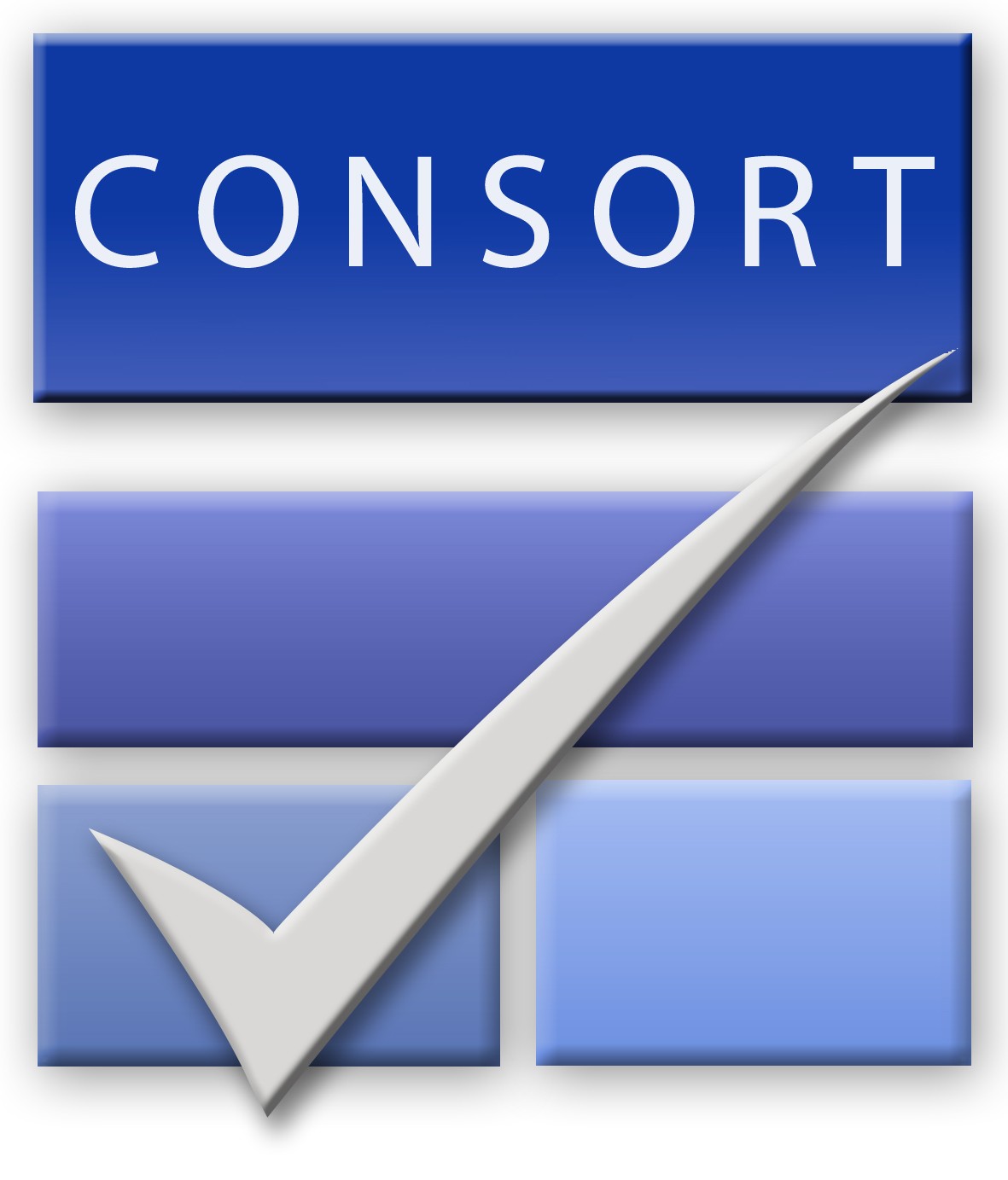
CONSORT 2010 checklist of information to include when reporting a randomised trial

| Section/Topic | Item No | Checklist item | | | Reported on page No |  |  |
| --- | --- | --- | --- | --- | --- | --- | --- |
| **Title and abstract** | | | | | |  |  |
|  | 1a | Identification as a randomised trial in the title | | | 1 |  |  |
|  | 1b | Structured summary of trial design, methods, results, and conclusions (for specific guidance see CONSORT for abstracts) | | | 2 |  |  |
| Introduction | | | | | |  |  |
| Background and objectives | 2a | Scientific background and explanation of rationale | | | 3 |  |  |
|  | 2b | Specific objectives or hypotheses | | | 3 |  |  |
| Methods | | | | | |  |  |
| Trial design | 3a | Description of trial design (such as parallel, factorial) including allocation ratio | | | 3 |  |  |
|  | 3b | Important changes to methods after trial commencement (such as eligibility criteria), with reasons | | | 4 |  |  |
| Participants | 4a | Eligibility criteria for participants | | | 4 |  |  |
|  | 4b | Settings and locations where the data were collected | | | 4 |  |  |
| Interventions | 5 | The interventions for each group with sufficient details to allow replication, including how and when they were actually administered | | | 5 |  |  |
| Outcomes | 6a | Completely defined pre-specified primary and secondary outcome measures, including how and when they were assessed | | | 7 |  |  |
|  | 6b | Any changes to trial outcomes after the trial commenced, with reasons | | | No changes (4) |  |  |
| Sample size | 7a | How sample size was determined | | | 7 |  |  |
|  | 7b | When applicable, explanation of any interim analyses and stopping guidelines | | | 7 |  |  |
| **Randomisation** |  |  | | |  |  |  |
| Sequence generation | 8a | Method used to generate the random allocation sequence | | | 4 |  |  |
|  | 8b | Type of randomisation; details of any restriction (such as blocking and block size) | | | 4 |  |  |
| Allocation concealment mechanism | 9 | Mechanism used to implement the random allocation sequence (such as sequentially numbered containers), describing any steps taken to conceal the sequence until interventions were assigned | | | 4 |  |  |
| Implementation | 10 | Who generated the random allocation sequence, who enrolled participants, and who assigned participants to interventions | | | 4 |  |  |
| Blinding | 11a | If done, who was blinded after assignment to interventions (for example, participants, care providers, those assessing outcomes) and how | | | 4 |  |  |
|  | 11b | If relevant, description of the similarity of interventions | | | 5 |  |  |
| Statistical methods | 12a | Statistical methods used to compare groups for primary and secondary outcomes | | | 7-8 |  |  |
|  | 12b | Methods for additional analyses, such as subgroup analyses and adjusted analyses | | | 7-8 |  |  |
| Results | | | | | |  |  |
| Participant flow (a diagram is strongly recommended) | 13a | For each group, the numbers of participants who were randomly assigned, received intended treatment, and were analysed for the primary outcome | | 9-10 | |  |  |
|  | 13b | For each group, losses and exclusions after randomisation, together with reasons | | 9-10 | |  |  |
| Recruitment | 14a | Dates defining the periods of recruitment and follow-up | | 9 | |  |  |
|  | 14b | Why the trial ended or was stopped | | Stopped as planned when the full pre-specified sample size was enrolled | |  |  |
| Baseline data | 15 | A table showing baseline demographic and clinical characteristics for each group | | 10-11 | |  |  |
| Numbers analysed | 16 | For each group, number of participants (denominator) included in each analysis and whether the analysis was by original assigned groups | | 9-10 | |  |  |
| Outcomes and estimation | 17a | For each primary and secondary outcome, results for each group, and the estimated effect size and its precision (such as 95% confidence interval) | | 12-14 | |  |  |
|  | 17b | For binary outcomes, presentation of both absolute and relative effect sizes is recommended | | 12-14 | |  |  |
| Ancillary analyses | 18 | Results of any other analyses performed, including subgroup analyses and adjusted analyses, distinguishing pre-specified from exploratory | | 15-16 | |  |  |
| Harms | 19 | All important harms or unintended effects in each group (for specific guidance see CONSORT for harms) | | NA | |  |  |
| Discussion | | | | | |  |  |
| Limitations | 20 | Trial limitations, addressing sources of potential bias, imprecision, and, if relevant, multiplicity of analyses | | | 18 |  |  |
| Generalisability | 21 | Generalisability (external validity, applicability) of the trial findings | | | 16 |  |  |
| Interpretation | 22 | Interpretation consistent with results, balancing benefits and harms, and considering other relevant evidence | | | 16 |  |  |
| Other information | | | | |  |  |  |
| Registration | 23 | Registration number and name of trial registry | | | Not registered (4) |  |  |
| Protocol | 24 | Where the full trial protocol can be accessed, if available | | | 4 |  |  |
| Funding | 25 | Sources of funding and other support (such as supply of drugs), role of funders | | | 20 |  |  |
|  |  | |  | | | |  |
